# Joint Effort towards Preventing Nutritional Deficiencies at the Extremes of Life during COVID-19

**DOI:** 10.3390/nu13051616

**Published:** 2021-05-12

**Authors:** Giulia C. I. Spolidoro, Domenico Azzolino, Raanan Shamir, Matteo Cesari, Carlo Agostoni

**Affiliations:** 1 Department of Clinical Sciences and Community Health, University of Milan, 20122 Milan, Italy; giulia.spolidoro@unimi.it (G.C.I.S.); domenico.azzolino@unimi.it (D.A.); macesari@gmail.com (M.C.); 2 Geriatric Unit, IRCCS Istituti Clinici Scientifici Maugeri, 20138 Milan, Italy; 3 Nutrition and Liver Diseases, Schneider Children’s Medical Center of Israel, Sackler Faculty of Medicine, Institute of Gastroenterology, Tel Aviv University, Tel Aviv 6997801, Israel; raanan@shamirmd.com; 4 Pediatric Intermediate Care Unit, Fondazione IRCCS Ca’ Granda Ospedale Maggiore Policlinico, 20122 Milan, Italy

**Keywords:** SARS-COV-2, malnutrition, sarcopenia, aging, nutrition, life-course, pediatrics, diet

## Abstract

The COVID-19 (Coronavirus disease 2019) pandemic is posing a threat to communities and healthcare systems worldwide. Malnutrition, in all its forms, may negatively impact the susceptibility and severity of SARS-CoV-2 (Severe Acute Respiratory Syndrome Coronavirus 2) infection in both children and older adults. Both undernutrition and obesity have been evoked as conditions associated with a higher susceptibility to the infection and poor prognosis. In turn, the COVID-19 infection may worsen the nutritional status through highly catabolic conditions, exposing individuals to the risk of malnutrition, muscle wasting, and nutritional deficiencies. Accordingly, the relationship between malnutrition and COVID-19 is likely to be bidirectional. Furthermore, the modification of nutritional behaviors and physical activity, required to limit the spread of the virus, are posing a challenge to health at both the extremes of life. Thus far, even the most advanced healthcare systems have failed to address the alarming consequences of malnutrition posed by this pandemic. If not properly addressed, we may run the risk that new and old generations will experience the consequences of COVID-19 related malnutrition.

## 1. Introduction

Since December 2019, the COVID-19 (Coronavirus disease 2019) pandemic is continuously threatening the sustainability of healthcare systems worldwide, with clinical manifestation ranging from asymptomatic to critical forms [1]. Especially at the beginning of the pandemic, this extreme situation has required the intervention of specialists coming from different backgrounds to address the shortage of medical personnel caring for all the infected subjects. Pediatricians and geriatricians have not been excluded and have worked together on this emergency in dedicated COVID-19 facilities. Therefore, despite the drama of this unprecedented event, the COVID-19 pandemic has had the positive side effect of bringing closer two specialties that are traditionally perceived as opposite. Indeed, although the pediatric and geriatric specialties are commonly seen in antithesis, they are less distant than what may be expected. The two extremes of life frequently share similar needs as both populations often require the presence of a caregiver. Furthermore, the two specialties may find potential interactions in people who are young in terms of chronological age but characterized by early biological aging (i.e., Down’s syndrome) [2]. In the context of COVID-19, pediatricians and geriatricians have had the unique opportunity to transform a theoretical virtual dialogue about finding common ground on which to construct a health alliance, to a practical application commanded by the healthcare emergency.

One topic of growing relevance for both the extremes of life during the pandemic is the relationship between the SARS-CoV-2 (Severe Acute Respiratory Syndrome Coronavirus 2) infection and malnutrition. COVID-19 may worsen nutritional status both directly, through a highly catabolic condition and the presence of gastrointestinal symptoms [3] and, indirectly, as a consequence of containment measures to limit the virus transmission. On the other hand, malnutrition itself may increase the susceptibility and severity of SARS-CoV-2 infection, with both undernutrition and overnutrition/obesity exerting a negative effect on the outcomes of the illness [4,5,6,7]. Accordingly, the relationship between COVID-19 and malnutrition is likely to be bidirectional.

Understanding the consequences that the combined action of COVID-19 and malnutrition might have at both extremes of life should be a priority for pediatrician and geriatrician. At the same time, elaborating a strategy to help addressing and properly managing the occurrence of the COVID-19-malnutrition duo should be prioritized. Therefore, the scope of this narrative review is first to provide an overview on the relationship between malnutrition and COVID-19 at the extremes of life and second to propose possible intervention and managing strategies that may help preventing or treating the occurrence of COVID-19-malnutrition.

We performed a narrative review to describe the existing literature on the bidirectional relationship between malnutrition and COVID-19 with special regard to the extremes of life. PubMed, EMBASE and Scopus databases were searched for relevant articles. We limited our search to manuscript published in the English language. Only when the pandemic will end, it will be possible to have a complete picture on the consequences of COVID-19 on nutritional status and vice versa.

## 2. Malnutrition at the Two Extremes of Life during the COVID-19

### 2.1. The Context

Pediatric and geriatric populations are vulnerable to malnutrition, which includes both under and overnutrition (i.e., obesity and overweight) [8], and this vulnerability relates to quantity as well as quality of the nutrient intake [2,9]. Regardless, in the context of COVID-19 pandemic nutritional status has been frequently overlooked. On the one hand, first, malnutrition could exacerbate the detrimental effects of the COVID-19 infection, as alterations in nutritional status are associated with a declined immune response and increased risk of infections, including viral infections, at both the extremes of life [10,11,12,13,14]. Second, the presence of malnutrition could determine high exposure to sources of damage, negatively affecting the repair and maintenance capacity for body systems [15]. On the other hand, COVID-19 itself can have a negative impact on nutritional status through several mechanisms including hyper-metabolism and increased energy requirements, as well as gastrointestinal symptoms (i.e., nausea, loss of taste and smell, vomiting, diarrhea) [16,17,18,19] which may lead to a decreased food intake [3]. Additionally, the COVID-19 pandemic has indirectly impacted nutritional balance both in developing as well as in developed societies, but with opposite negative outcomes. In Third World countries, containment measures (i.e., lockdown, social isolation, and physical distancing) have resulted in a break in the food chain, increasing undernutrition and social inequalities. Oppositely, in the “prosperous” Western societies, the same approach has created an obesogenic environment characterized by a reduction in physical activity (in favor of a sedentary lifestyle), as well as by an increase in convenience foods and alcohol consumption, along with a decreased consumption of fruit and vegetables [20,21,22]. Despite the high caloric dietary intake, the shift to a qualitatively unhealthy diet, characterized by a high amount of saturated fats and refined carbohydrates including simple sugars on the one hand, and a low content of fiber, antioxidants, and unsaturated fatty acids on the other, might then expose individuals to nutritional inadequacies or deficiencies, thus increasing the risk of developing obesity and type 2 diabetes, which are in turn associated with negative COVID-19 outcomes [23].

By altering nutritional status, COVID-19 infection may negatively impact the accumulation of biological reserves by young individuals, that will determine the peak capacity for a body system and the rate of the subsequent decline during later life [24]. Instead, in older people malnutrition and COVID-19 may have immediate detrimental effects, depriving the already decreased biological capital of the aged individual.

### 2.2. Older Persons

As mentioned above, COVID-19 infection may worsen one’s nutritional status in several ways, both directly and indirectly. Direct effects of the COVID-19 pandemic are most evident in the older population. In addition to the respiratory tract, the gastrointestinal system may also be affected by SARS-CoV-2 infection with nausea, diarrhea, vomiting, and anorexia [25,26,27,28,29,30,31] (Table 1).

Additionally, common symptoms of COVID-19 also include anosmia (loss of smell) and ageusia (loss of taste) [1], which are acknowledged to cause anorexia in older people [43]. Anorexia may also be secondary to the elevated levels of inflammatory cytokines observed in COVID-19 infection [27]. In COVID-19 patients, a highly catabolic state resulting from the augmented inflammatory response may also lead to skeletal muscle wasting [27,44]. Therefore, it becomes evident how COVID-19 can alter several physiological conditions increasing the risk of undernutrition (Table 1). During the hospital stay, various conditions (i.e., comorbidities, inflammatory states, and infections) may worsen nutritional status [45,46,47]. For instance, prolonged immobilization during hospitalization due to COVID-19 may further worsen muscle loss [48]. It has been reported that at least one-third of patients presents malnutrition at hospital admission [10], with higher rates seen in older people [49]. Indeed, nearly one third of patients who were not malnourished at the time of admission may have developed malnutrition during hospitalization [50].

As stated above, it is widely reported that also obesity increases COVID-19 susceptibility and disease severity and mortality (Table 1) [1,51]. Obesity and related comorbidities could contribute to the worst outcomes of COVID-19 through several mechanisms. First, obesity is characterized by a chronic pro-inflammatory state, which could further feed the so-called “cytokine storm” contributing to the most severe consequences of the infection [52,53]. Second, obesity is also associated with an impaired immune response and negatively affects respiratory function [5,53]. Third, it has been noted an elevated expression of the ACE-2 (angiotensin-converting enzyme 2), which is responsible for the entry of SARS-CoV-2 into target cells, in the adipose tissue, potentially explaining the higher susceptibility to the infection and the disease severity seen in obese patients [54,55]. Finally, the pro-coagulant profile associated with obesity may promote thromboembolic complications in COVID-19 patients [56]. Of note, obesity has been also associated with micronutrient deficiencies [57], probably through the mediation of chronic inflammation and micronutrient-poor diets [58]. Obesity status may be frequently underestimated in older adults who can present with increased adiposity alongside with muscle mass decline. In fact, an excessive body fat accumulation may be present in those older people considered normal or overweight according to BMI, characterizing the so-called sarcopenic obesity [59,60]. The combination of sarcopenia and obesity is even more detrimental since it seems to feed an additional production of inflammatory mediators, creating a vicious circle characterized by a further (accelerated) muscle catabolism and additional weight gain [61,62].

The negative effects of the COVID-19 pandemic on nutritional status may be found also outside the acute care setting. Containment measures to limit the spread of the virus may also threaten nutritional status in several ways. Such drastic measures can result in limited access to preventive care and nutritional counseling [63,64]. Confinement may determine the change of eating behaviors and physical activity, with negative effects on mental health [64,65]. Access to fresh and healthy food products may be limited [66,67], especially in those people with a poor socioeconomic status or mobility disability [68]. For instance, older adults may experience a limited access to grocery shopping and reduced support for shopping and cooking [22]. Some people may, therefore, rely on delivery services, visiting stores less frequently [68]. Finally, the decrease in physical activity due to confinement may accelerate the loss of muscle mass and strength in older people [27].

### 2.3. Infants and Children

Even though it is acceptable that, at the beginning of the COVID-19 pandemic, healthcare and social systems have found objective difficulties in keeping up with the already standing nutritional healthcare and educational programs, as well as in addressing the new challenge of taking care of the nutritional aspect during a pandemic, the necessity to address and promptly treat COVID-19 related malnutrition and nutritional deficiencies is now widely recognized.

Several factors are undermining nutrition adequacy in children, especially in low- and middle-income countries. The humanitarian programs have been slowed down during the COVID-19 pandemic, determining limited access to clean water and nutritious food, along with the interruption of healthcare services addressing the problem of protein-energy malnutrition from early life [63]. According to projection models developed to predict the impact of COVID-19 on the healthcare and economic system of low- and middle-income countries, the number of cases of acute malnutrition (wasting) in 2020 was expected to raise up to 50%, along with an increase in maternal and child additional death [69,70]. However, the validity of these past projections has yet to be confirmed.

The COVID-19 pandemic is also predisposing to other malnutrition related conditions such as stunting (chronic malnutrition), micronutrient deficiencies, and overweight [71]. Furthermore, COVID-19 has raised the issue of food insecurity even in developed and wealthy countries. To low-income families that had so far relied on the school system for at least one meal per day, the collapse of the social educational system in the wake of the COVID-19 pandemic represents a big challenge. Moreover, many families have lost at least one wage, adding economic insecurity to the already standing social pressure [63,68]. On the other hand, several surveys carried out during national lockdowns in Western countries have also pointed out that the detrimental effects of the COVID-19 pandemic may also explicate with the increasing sedentary lifestyles, as well as with the indulgence in poor diet choices (increased junk food consumption accompanied to scarce adherence to healthy diet), especially among the youngest [72]. Thus, COVID-19 may also negatively affect children’s nutritional status and healthiness by creating an obesogenic environment [73], consequently increasing the prevalence of overweight and obesity in children [74]. The negative impact of SARS-CoV-2 is, therefore, undeniable as it may pose a threat to the programs aiming to educate on nutrition and to prevent both under- and overnutrition [14,63].

Malnutrition may lead not only to acute negative effects on the individuals, but may also add medium- and long-term negative effects on healthiness and/or the course of diseases in later life. This is true especially for the pediatric population, since children require energy to support the costs of growth. Consequently, the presence of nutritional unbalances may seriously alter development, growth potential and body composition, with permanent consequences [72]. Recently, Finch et al. [75] compared the potential long term negative effects of COVID-19 related malnutrition to the so-called “Hongerwinter”, an historical period during the Nazi German occupation of the Netherlands, during which the food rations were so poor that the population lived a year-long starvation. In the years and decade that followed, it was observed that children born to mothers’ who suffered famine during pregnancy were at higher risk of developing chronic conditions such as cardiovascular diseases, obesity and diabetes. Such observations support the evidence that environmental factors occurring as early as in utero may have a significant influence on health outcomes during the life-course. This theory was first proposed by Hales and Barker with the name of “thrifty phenotype” [76], and represents the starting point for the “first 1000 days” related claims and, the “maternal and early life programming” movement [2,77]. These different points integrate each other, leading to the conclusion that several factors including nutrition and stress, as well as parental health status, may influence the health status of future generation of population who are living the COVID-19 pandemic. An overview of the main studies exploring the bidirectional relationships between malnutrition and COVID-19 in children and young individuals is presented in Table 2.

## 3. Management of Nutritional Status at the Two Extremes of Life during COVID-19

An early identification of risk or presence of malnutrition is pivotal even in normal times. Therefore, it is crucial to assess the individual nutritional status in all subjects admitted to the hospital, even more during the COVID-19 pandemic. Untreated malnutrition is associated with longer hospital stay and higher risk of comorbidities, which may seriously impair patients’ body functions and quality of life, as well as survival rates [40,78]. Also at the community level, malnutrition and nutritional risks may have long-term consequences on health and body functions. Prevention and treatment of malnutrition and nutritional deficiencies may impact the prognostic outcomes at any level and in any stage of life. Through shared genetic and environmental factors (the epigenetic phenomena), nutritional problems can span generations creating a vicious cycle of malnutrition, negatively affecting body composition and health status throughout the life-course, and indirectly posing a risk of developing non-communicable diseases in later life [58,79]. Intervening through programs that simultaneously involve the entire family household, from grandparents to grandchildren, may therefore represent a strong strategy to interrupt the so-called “intergenerational cycle of malnutrition” [79] and should be pursued by both geriatricians and pediatricians.

Several screening tools are available to identify people at risk of malnutrition or already malnourished. However, implementing these during the COVID-19 pandemic is challenging. Rapid instruments can help clinicians assessing nutritional status. To date, there might be difficulties to retrieve information directly from patients about recent dietary intake or weight changes because of health problems (i.e., severe respiratory conditions, cognitive decline, low level of consciousness) [80]. It must be recognized that it may be difficult to obtain information from caregivers or relatives because of the limited access to the hospitals. Video conferencing aids may help retrieve information about nutrition from relatives or caregivers (i.e., weight loss, reduced dietary intake) [81]. The utilization of even simple instruments such as scales and/or stadiometers may be difficult during this emergency circumstance for various reasons (i.e., unavailability in COVID+ facilities, hygiene reasons, containment measures) [82]. In such cases, clinicians should still consider self-reported or estimated values. Additionally, the most accurate techniques to assess body composition (i.e., dual-energy X-ray absorptiometry, bioelectrical impedance analysis, magnetic resonance imaging, computed tomography) may not be available in most settings or of difficult implementation during the COVID-19 pandemic. Indeed, alternative measures such as mid-arm muscle or calf circumference, even if less accurate, should be considered in older subjects [81]. As for pediatrics, there is a vast choice of growth charts or standards available for clinical practice: international, national, for healthy subjects, or even disease-specific. Generally, it is suggested to use the official WHO growth standards, developed to differentiate infant’s growth according to the early type of feeding in the first years of life [83]. Most recently, the United Nations Children’s Fund (UNICEF) has proposed that the risk of undernutrition in children may be monitored directly by caregivers through the use of a user-friendly mid upper arm circumference (MUAC) tape, thus decreasing the risk of exposure to COVID-19 by reducing health center visits [84]. Although the validation of the UNICEF MUAC tape is still to be ascertain, the innovative design of this new tool provides caregivers with a safe way to monitor the child’s nutritional status even during the COVID-19 emergency.

A step forward in the monitoring of nutritional issues and muscle loss has been the proposal of a new app named Remote-Malnutrition APP (R-MAPP) [85]. This application has been recently developed to remotely identify older people at risk of malnutrition and sarcopenia during the COVID-19 pandemic. R-MAPP includes two validated questionnaires: The Malnutrition Universal Screening Tool (MUST) and the Strength, Assistance with walking, Rise from a chair, Climb stairs, and Falls (SARC-F) to rapidly screen for malnutrition and sarcopenia, respectively. Given that both pediatric and geriatric population may share similar challenges regarding nutritional imbalances during this pandemic, the development of an application similar to R-MAPP but aimed at assessing nutritional status in the entire household environment, may represent a good starting point to address the negative consequences on nutritional status posed by the COVID-19 pandemic. Of course, when developing an “all age inclusive” R-MAPP, attention should be paid to the fact that neither MUST nor SARC-F are validated for the pediatric population, therefore, it would be necessary to use different screening tools to assess nutritional status in the youngest. Malnutrition screening tools specifically designed for the pediatric community setting, such as the Electronic Kids Index (E-KINDEX) or the Nutrition Screening Tool for Every Preschooler (NutriSTEP), may be employed. However, a recent survey pointed out that community setting specific malnutrition screening tools are not as accurate [86]. Therefore, for the purpose of remote screening of malnutrition during the pandemic, developing a different tool may be preferable, perhaps adapting the more accurate malnutrition screening tools validated for the hospital setting.

In conclusion, the ideal remote malnutrition screening app should specifically investigate unintentional weight loss/gain, changes in eating behaviors, presence of underweight or overweight in subjects of all ages, with reliable age-specific nutritional screening tools. The app should also include a complementary (not compulsory) section for the subjects to provide current weight and height information (thus allowing the app to compute nutritional-relevant body composition indexes). Finally, the app should specifically address growth in pediatric subjects, as well as provide a dedicated section to investigate the presence of sarcopenia (i.e., through the SARC-F score) in older subjects. The use of new technologies such as smartphones “apps” may also be considered to deliver physical exercise designed to include also activities that are targeted to both the youngest and the oldest populations. However, particular care should be paid when trying to apply such technologies at the extremes of life. The use of smartphone in children may be criticized as it is recognized that longer screen-time in the young population has negative effects, including (paradoxically) increased risk of developing obesity. Oppositely, older people may experience difficulties in understanding basic functioning of smartphones, let alone mastering the download and understanding of medical apps. In this sense, pediatricians and geriatricians should join effort by finding ways to implement the use of new technologies without disrupting the fragile environment of our youngest and oldest generations. Once again, this could be achieved through the mediation of a caregiver, but more extensively, it could represent an occasion to develop interactive digital tools that promote the monitoring of nutritional status and the implementation of nutritional strategies for the entire household (grandparents, parents, and grandchildren), shifting the standard of care from a diseased-focus system to a life-course approach.

Protein deficiency needs to be prevented at both the extremes of life, especially in COVID-19. Older adults need at least 1.0 g/kg of body weight/day to prevent muscle loss. The protein intake should be increased to 1.2–1.5 g/kg of body weight/day in the presence of acute or chronic diseases. In severe forms of COVID-19, characterized by high catabolic processes such as wasting syndrome, the intake of proteins may be increased up to 2.0 g/kg of body weight/day [55,56,57]. In children, the prevention of acute protein-energy malnutrition requires different intakes, according to weight, age, and severity of the disease. To date, there is no specific indication for the management of ICU admitted pediatric patients with a diagnosis of COVID-19, but the standard guidelines for nutritional support in PICUs are valid tools to direct the nutritional support in this category [87,88,89]. In addition to protein intake, it is mandatory an adequate caloric provision since if energy intake is not sufficient to meet demands, body fat, and muscle are catabolized to provide energy [90]. Determining caloric needs is best based on direct measurement of resting energy expenditure [91]. However, a driving value for energy intake of 27–30 Kcal/kg of body weight/day can be recommended in older people [81] and the Schofield equation can be used in children [91]. Both energy and protein intakes should be adjusted to nutritional status, disease status, pre-illness physical activity level, and preferences [92]. During hospitalization, nutritional status may also be assessed by measuring specific nutritional-relevant biomarkers. Visceral proteins such as albumin, pre-albumin, retinol binding protein (RBP), and transferrin are especially useful to detect eventual alterations in the protein pool. Specifically, both RBP and pre-albumin have a short half-life (12 h and 2–3 days, respectively), therefore, a reduction in the levels of these two proteins may indicate acute nutritional status changes. Conversely, the monitoring of protein with longer half-life, such as transferrin and albumin, may help highlighting chronic changes in nutritional status. Finally, among the blood values that are less likely to be measured routinely, IGF-1 is considered a useful parameter for early detection of protein-energy malnutrition [93]. All these protein-energy markers are useful to complement the clinical nutritional assessment. Indeed, no single marker can assess overall nutritional status given that each of these has its own limitations especially in the acute phase response and in other disease conditions (i.e., renal and liver failure). However, the levels of these biomarkers may be of interest in providing information about catabolic and/or anabolic conditions [94]. Given that the vast majority of these biomarkers are influenced by inflammatory states, the clinical interpretation of their levels along with the inclusion of an inflammatory marker (i.e., C-reactive protein) should, therefore, be recommended.

Additional information on protein synthesis/anabolism and protein turnover/catabolism can be gathered by the monitoring of urine creatinine and urinary nitrogen excretion. Other useful biochemical markers for the assessment of nutritional status include hemoglobin, total protein, serum cholesterol and blood lymphocyte count, as well as the evaluation of specific micronutrients deficits (iodine, folate, vitamins, and other essential micronutrients in general) [93,95]. Particular attention should be paid to the refeeding syndrome in patients who are severely malnourished since the target of energy intake should be gradually achieved and monitored carefully in these cases. In those subjects who fail to ingest adequate amounts of energy and nutrients with natural foods, multi-nutrient formulas providing both macro- and micronutrients should be considered [96]. If it is not possible to meet nutritional demands per os, as in the case of COVID-19 patients requiring mechanical ventilation, enteral nutrition should be initiated as early as possible to reduce complications [97]. Given the risk of refeeding syndrome, it is also recommended close monitoring of serum levels of phosphate, magnesium, potassium, and thiamine during the first three days after enteral or parenteral nutrition, which should be promptly supplemented in case of even mild deficiencies [92]. The nutritional status should be mandatorily addressed upon admission within a hospital setting, either COVID-19 related or not. For instance, several studies have now suggested a link between vitamin D deficiency and immune system dysfunction in patients with COVID-19 [98]. It has also been suggested that an adequate micronutrients nutritional status may help in preventing viral infections and severity of illness [99]. Some vitamins (i.e., vitamin A, B6, B12, D, E) and minerals like as zinc and selenium seem to promote a correct immune function [100]. Therefore, low levels of these micronutrients have been correlated with negative clinical outcomes in viral infections. Several studies suggested an association between low levels of vitamin D and worst outcomes in COVID-19 patients [101,102,103,104,105]. Furthermore, in the last decade vitamin D deficiency has been associated with some other viral conditions like as influenza, human immunodeficiency virus and hepatitis C [81]. However, some authors have questioned an association with influenza [106,107]. It has been also observed that COVID-19 infection may be associated to the risk of developing hypocalcemia and hypomagnesemia [108]. To date, the antioxidant properties of magnesium as well as its role as an inhibitor of the release of inflammatory cytokines, are well recognized [109]. However, low levels of other micronutrients (i.e., vitamin A, E, some B vitamins, zinc, and selenium) have also been suggested to play a role in mediating negative outcomes during viral infections [81]. Indeed, a close monitoring of the levels of these compounds is generally recommended. Addressing micronutrients deficiencies in COVID-19 patients may result in better outcomes for infected subjects. Many studies are investigating the potentials of providing micronutrients supplements to help busting up the immune system, thus ameliorating the immune response to SARS-CoV-2 infection. To date, only a few of these studies have already been published, while a larger number has yet to finalize the enrolment phase of the clinical trial. In a study from Tan et al., the authors report that COVID-19 infected subjects older than 50 years who received a combination of vitamin D, magnesium and vitamin B12 during hospitalization were less likely to require oxygen therapy or intensive care support as compared to subjects who did not receive the supplements [110]. Regarding vitamin D, Annweiler et al. [111] report that regular bolus vitamin D supplementation previous to COVID-19 infection was linked to less severe COVID-19 symptoms and better survival in infected older subjects, but no significant difference in illness outcomes was observed when bolus supplementation was initiated during hospitalization. Similarly, Murai et al. [112] observed no significant reduction on length of stay among COVID-19 hospitalized patients who were administrated a single high dose of vitamin D3, compared with placebo. On contrary, a pilot study in Spain determined that the administration of high dose vitamin D at early admission and during the first seven days of hospitalization significantly reduced ICU admissions in 50 hospitalized COVID-19 treated with hydroxychloroquine and azithromycin versus hydroxychloroquine alone [113]. Likewise, Ling et al. [114], found that patient who received vitamin D supplementation in the first 7 days of hospitalization had a reduced risk of COVID-19 mortality. Concerning zinc supplementation, in a study from Carlucci et al. [115], it was observed that COVID-19 patients treated with hydroxychloroquine and azithromycin who were also supplemented with zinc sulphate had lower mortality or transfer to hospice rate, as well as an overall increase frequency of being discharged home compared to subjects who were treated with hydroxychloroquine and azithromycin alone. However, a smaller study investigating the association between zinc supplementation and survival of hospitalized patients did not found any difference [116]. Finally, regarding vitamin C, there is currently many trials investigating its potential role in ameliorating COVID-19 outcomes [117]. Initial results published by Zhang et al. [118], indicated a promising reduction of 28-day mortality in subjects treated with intravenous vitamin C for 7 days. Further evidence on the positive effects of macronutrients supplementation on COVID-19 outcomes might emerge from the ongoing clinical trials currently investigating the topic. An updated list of the current trials investigating the topic was provided by Di Matteo et al., in a review paper investigating food potentials in influencing COVID-19 outcomes [119]. Other elements that are currently being investigated for their immuno-modulatory action are polyunsaturated fatty acids (PUFA) and probiotics, as recently reported by Lordan et al. [120].

However, despite the importance of preventing and treating micronutrient deficiencies, there is no evidence that micronutrient supplementation in non-deficient subjects would be protecting against COVID-19 or improving clinical outcomes of the infection. Indeed, the European Society for Clinical Nutrition and Metabolism recommends an intake of vitamins and minerals according to daily allowances in order to maximize general anti-infection nutritional defense [81].

## 4. Conclusions and Future Perspectives

An impaired nutrition has been associated with an adverse outcome for both COVID-19 susceptibility and disease severity, leading to worst outcomes in the geriatric population. On the other hand, the negative effects of the pandemic itself (social isolation, containment measures, school closure, food insecurity) are threatening nutritional status, creating a vicious circle within the infant and pediatric population as well as in old people. An innovative educational program involving together pediatricians and geriatricians may represent a strategy to move on global nutritional issues, at a public health level, as well as in the clinical setting, combating together in transforming the negative impact of the unexpected COVID-19 pandemics. Such a strategy should include also newer electronic tools to assess nutritional status and nutritional needs remotely (i.e., delivery of nutritional counselling via videoconferencing, monitoring nutritional status with ad hoc designed apps). Pediatricians and geriatricians should collaborate to redesign classic nutritional scores and nutritional assessment tools in a more user-friendly perspective unifying problems and issues raised by the two fragile (and sensitive!) extremes of life.

## Figures and Tables

**Table 1:** Overview of the main studies exploring the relationships between malnutrition and COVID-19 in adults and older adults.

Reference	Study Design and Sample	Aim	Relevant Results
**Gastrointestinal symptoms/Anorexia**			
Pan et al., 2020 [28]	Cross-sectional study; 204 COVID-19 patients; mean age 52.9 (SD 16) years	Investigate the prevalence and outcomes of COVID-19 patients with digestive symptoms.	103 patients (50.5%) reported digestive symptoms, including lack of appetite (81 [78.6%] cases), diarrhea (35 [34%] cases), vomiting (4 [3.9%] cases), and abdominal pain (2 [1.9%] cases).
Zheng et al., 2020 [29]	Cross-sectional study; 1320 patients; median age 50 (IQR 40–57) years.	Compare clinical characteristics and outcomes between patients with and without GI symptoms.	192 patients (14.5%) reported gastrointestinal symptoms, including diarrhea (107 [55.7%] cases), abdominal pain (11 [5.7%] cases), anorexia (62 [32.3%] cases), nausea and vomiting (57 [29.7%] cases).
Redd et al., 2020 [30]	Multicenter cohort study; 318 patients; mean age 63.4 (SD 16.6) years.	Examine prevalence and features of GI manifestations associated with SARS-CoV-2 infection	61.3% of patients reported at least 1 gastrointestinal symptom on presentation, most commonly loss of appetite (34.8%), diarrhea (33.7%), and nausea (26.4%).
Meng et al., 2020 [31]	Review	Assess the relationship between olfactory dysfunction and COVID-19.	Anosmia ranged from 33.9 to 68% with female dominance.
Parasa et al., 2020 [25]	Systematic review and meta-analysis of 23 published and 6 preprint studies; 4805 patients; mean age 52.2 (SD 14.8) years	Examine incidence rates of gastrointestinal symptoms among patients with COVID-19 infection.	12% of patients with COVID-19 infection reported gastrointestinal symptoms, including diarrhea (7.4%), nausea, and vomiting (4.6%).
**Undernutrition**			
Bedock et al., 2020 [3]	Observational longitudinal study; 114 COVID-19 patients, mean age 59.9 (SD 15.9) years.	Examine the association between malnutrition and disease severity at admission and the impact of malnutrition on clinical outcomes (i.e., ICU transfer or death).	The overall prevalence of malnutrition was 42.1% (moderate: 23.7%, severe: 18.4%). The prevalence of malnutrition reached 66.7% in patients admitted from ICU.
Rouget et al., 2020 [24]	Prospective observational cohort study; 80 COVID-19 patients; median age 59.5 (IQR 49.5–68.5).	Evaluate the prevalence of malnutritionin patients hospitalized for COVID-19.	The prevalence of malnutrition was 37.5% with 26% of hospitalized patients who presented severe malnutrition.
Li et al., 2020 [32]	Cross-sectional study; 182 COVID-19 older patients; mean age 68.5 (SD 8.8) years.	Investigate the prevalence of malnutrition and its related factors in older patients with COVID-19.	96 patients (52.7%) were malnourished and 50 patients (27.5%) were at risk of malnutrition
Yu et al., 2020 [33]	Retrospective survey study; 139 patients with COVID-19; mean age 61.47 (SD 14.76) years.	Examine the association of malnutrition with duration of hospitalization in patients with COVID-19.	75 patients had nutritional risk (53.96%). Compared with the patients in the normal nutrition group, the hospitalization time was longer (15.67 [SD 6.26] days versus 27.48 [SD 5.04] days, *p* = 0.001)
Allard et al., 2020 [34]	Retrospective study; 108 COVID-19 patients; mean age 61.8 (SD 15.8).	Determine the percentage of malnutrition and its prognosis in patients admitted for COVID-19.	42 (38.9%) patients were malnourished. Moderate or severe nutritional risk was found in 83 (84.7%) patients. Malnutrition was not associated with COVID-19 severity, while nutritional risk was associated with severe COVID-19 (*p* < 0.01).
**Obesity**			
Suleyman et al., 2020 [35]	Case series; 463 patients with COVID-19; mean age 57.5 (SD 16.8) years	Describe the clinical characteristics and outcomes of patients with COVID-19 infection.	Severe obesity (i.e., BMI ≥ 40) was independently associated with intensive care unit admission (OR: 2.0; 95% CI: 1.4–3.6; *p* = 0.02)
Petrilli et al., 2020 [36]	Prospective cohort study; 5279 COVID-19 patients; median age 54 (IQR 38–66) years.	Examine outcomes of people admitted to hospital with COVID-19.	Any increase in BMI (i.e., BMI > 40) was strongly associated with hospital admission (OR: 2.5; CI: 1.8–3.4; average marginal effect: 14%)
Simonnet et al., 2020 [37]	Retrospective cohort study; 124 COVID-19 patients admitted in ICU; median age 60 (IQR 51–70) years.	Analyze the relationship between clinical characteristics, including BMI, and the requirement for invasive mechanical ventilation.	Obesity (BMI > 30 kg/m^2^) and severe obesity (BMI > 35 kg/m^2^) were present in 47.6% and 28.2% of cases, respectively. The proportion of patients who required IMV increased with BMI categories (*p* < 0.01, Chi square test for trend)
Hajifathalian et al., 2020 [38]	Retrospective review; 770 COVID-19 patients; mean age of 63.5 (SD 17) years	Examine the role of obesity in the clinical course of COVID-19 patients.	Obese patients were more likely to present with fever, cough and shortness of breath. Obesity was also associated with a significantly higher rate of ICU admission or death (RR = 1.58, *p* = 0.002)
Busetto et al., 2020 [39]	Retrospective cohort study; 92 COVID-19 patients; mean age 70.5 (SD 13.3) years	Assess the relationship between the severity of COVID-19 and obesity classes according to BMI.	A higher need for assisted ventilation and a higher admission to intensive or semi-intensive care units were observed in patients with overweight and obesity (*p* < 0.01 and *p* < 0.05, respectively)
Malik et al., 2021 [40]	Meta-analysis of 14 studies; 10, 233 confirmed COVID-19 patients;	Assess the effect of obesity on outcomes in the COVID-19 hospitalizations.	The overall prevalence of obesity was 33.9% (3473/10,233). COVID-19 patient with obesity had higher odds of poor outcomes (OR: 1.88; 95% CI: 1.25–2.80; *p* = 0.002).
Ho et al., 2020 [41]	Systematic Review and Meta-analysis of 61 studies; 270, 241 patients.	Examine the relationship between COVID-19 and obesity.	The pooled prevalence of obesity was 27.6% (95% CI: 22.0–33.2). Obesity was not significantly associated with increased ICU admission or critical illness (OR: 1.25, 95% CI: 0.99–1.58, *p* = 0.062) but was significantly associated with more severe disease (OR: 3.13, 95% CI: 1.41–6.92, *p* = 0.005), mortality (OR: 1.36, 95% CI: 1.09–1.69, *p* = 0.006) and a positive COVID-19 test (OR: 1.50, 95% CI: 1.25–1.81, *p* < 0.001).
Huang et al., 2020 [42]	Systematic review and meta-analysis of 33 studies (30 studies defined obesity via BMI and 3 studies using VAT adiposity); 45, 650 subjects.	Investigate the effects of obesity with the risk of severe disease among patients with COVID-19.	Higher BMI was associated with severe COVID-19 (OR 1.67, 95% CI: 1.43–1.96; *p* < 0.001), hospitalization (OR 1.76; 95% CI: 1.21–2.56, *p* = 0.003), ICU admission (OR 1.67, 95% CI: 1.26–2.21, *p* < 0.001), IMV requirement (OR: 2.19, 95% CI: 1.56–3.07, *p* < 0.001), and death (OR 1.37, 95% CI: 1.06–1.75, *p* = 0.014). Severe COVID-19 cases showed significantly higher VAT (SMD: 0.50, 95% CI: 0.33–0.68, *p* < 0.001), hospitalization (SMD: 0.49, 95% CI: 0.11–0.87; *p* = 0.011), ICU admission (SMD: 0.57, 95% CI: 0.33–0.81; *p* < 0.001) and IMV support (SMD: 0.37, 95% CI: 0.03–0.71; *p* = 0.035).

GI = Gastrointestinal; SD = Standard deviation; IQR = interquartile range; OR = Odds ratio; RR = risk ratio; CI = confidence interval; ICU = intensive care unit; BMI = body mass index; IMV = invasive mechanical ventilation; VAT = visceral adipose tissue; SMD = standardized mean difference.

**Table 2:** Overview of the main studies exploring the relationships between malnutrition and COVID-19 in children and young individuals.

Reference	Study Design and Sample	Aim	Relevant Results
**Gastrointestinal symptoms**			
Lu et al., 2020 [16]	Observational study; 171 children with COVID-19; median age 6.7 years (range 1 day–15 years)	Describe the epidemiologic characteristics, clinical features, and radiologic findings of children with COVID-19.	Children had a milder clinical course compared to adults. GI symptoms were not very common in children. 15 patients presented diarrhea (8.8%) and 11 (6.4%) vomiting.
Garazzino et al., 2020 [17]	Observational multicentre study; 168 children with COVID-19.	Collect preliminary data on COVID-19 presentation in children	In children, GI symptoms were frequent (18%).
Giacomet et al., 2020 [18]	Observational retrospective multicentre study; 127 children with COVID-19	Explore the presence of GI symptoms in children with COVID-19 and the potential correlation between GI symptoms and severity of illness	GI symptoms were present in 28.3% of the children enrolled. COVID-19 severity was positively correlated with the presence of GI symptoms.
**Undernutrition**			
Akseer et al., 2020 [63]	Review	Identify main risk factors for maternal and child undernutrition during the COVID-19 pandemic and provide guidance to reduce the consequent undernutrition	Children and mothers’ risk of undernutrition may be increase during the pandemic due to food insecurity/poor diet quality, reduced income/limited financial resources, restricted health services, interrupted education, unhealthy household environment.
Headey et al., 2020 [69]	Global health projection study	Provide an overview on the impact of COVID-19 on childhood malnutrition and nutrition-related mortality using three different projection models.	Low- and middle-income countries are expected to have an average 7.9% decrease in the gross national income, which might associate to an increase in moderate to severe wasting (chronic malnutrition) in children (up to 14.3%). Together with a projected year average reduction in nutrition and health services coverage of about 25% such event may lead to about 128,605 additional death in children <5 years during 2020.
Roberton et al., 2020 [70]	Global health projection study	Estimate the additional child (<5 years) and maternal deaths resulting from potential health systems disruption and decreased access to food.	A reduction by 9.8–51.9% of the coverage of essential maternal and child health interventions might result in increased prevalence of wasting by 10–50% and additional child and maternal death in 2020.
**Obesity/Overweight**			
Nogueira-de-Almeida et al., 2020 [20]	Clinical review	Examine the factors contributing to increased COVID-19 susceptibility and severity in obese children and adolescents.	Obesity related risk factors such as chronic subclinical inflammation, impaired immune response, and association with communicable diseases may explain the increased evidence of higher severity and mortality rate for COVID-19 in the adult as well as in the young population.
Storz, 2020 [73]	Review	Present supporting evidence that the COVID-19 pandemic will aggravate the childhood obesity	Through multiple factors (lockdown and movement restrictions, quarantine, home-confinement, and social distancing, school closures, pandemic insecurity and economic hardship) COVID-19 will create an obesogenic environment, increasing childhood obesity
Browne et al., 2020 [14]	Report	Address the impact of COVID-19 on children with obesity and propose potential interventions to reduce the negative outcome.	Children with obesity may face biopsychosocial risks during COVID-19, which may lead to stress and consequent impaired inflammation and immune response to COVID-19 Access to timely, comprehensive healthcare is critical during the pandemic.
Leon-Abarca, 2020 [4]	Observational study; 21,161 subjects under 18 years old	Identify risk factors and pre-existing conditions associated with COVID-19 illness in childhood.	Obesity (3.1%) was among the most common pre-existing condition in children with COVID-19. Children with obesity had 4.5-fold probability of presenting pneumonia and 2.5-fold probability of being hospitalized.
Kass et al., 2020 [5]	Observational study; 265 COVID-19 patients admitted to hospital	Investigate the correlation between BMI and age in COVID-19 patients admitted to the ICU	Significant inverse correlation between age and BMI was observed, suggesting that younger individuals with COVID-19 admitted to hospital and those requiring ICU support are more likely to be obese.
Zhang et al., 2020 [6]	Observational retrospective study; 53 young patients (20 to 45 years).	Examine the risk factors of mortality in young patients with COVID-19 with specific attention to the relationships between obesity and COVID-19 mortality.	In young patients, obesity (high BMI) was strongly associated with high risk of mortality for SARS-CoV-2 infection. In addition, aggravated inflammatory response, enhanced cardiac injury and increased coagulation activity were also reported as contributing mechanism to the high mortality, compared to the COVID-19 survivor counterpart.
Deng et al., 2020 [7]	Observational retrospective study; 65 COVID-19 hospitalized patients aged 18 to 40 years	Explore the indicators for COVID-19 severity in young patients aged 18 to 40 years.	In young adults, severe COVID-19 cases had higher BMI compared to moderate cases (average 29.23 vs. 22.79 kg/m^2^, *p* < 0.01).
An R., 2020 [74]	National health projection study	Project the impact of the COVID-19 pandemic on childhood obesity by simulating the BMI z-score trajectory of a representative cohort under a control scenario without COVID-19 or under 4 alternative scenarios with COVID-19.	Relative to the control scenario without COVID-19, scenarios 1, 2, 3, and 4 were associated with an increase in the mean BMI z-score

GI = Gastrointestinal; ICU = intensive care unit; BMI = body mass index.
